# Heat stress modulates differential response in skin fibroblast cells of native cattle (*Bos indicus*) and riverine buffaloes (*Bubalus bubalis*)

**DOI:** 10.1042/BSR20191544

**Published:** 2020-02-11

**Authors:** Umesh K. Shandilya, Ankita Sharma, Monika Sodhi, Manishi Mukesh

**Affiliations:** Animal Biotechnology Division, ICAR-National Bureau of Animal Genetic Resources, Karnal 132001, Haryana, India

**Keywords:** Bos indicus cattle, cellular resposne, heat stress, Riverine buffaloes, skin fibroblast, transcriptional respsone

## Abstract

Heat stress in hot climates is a major cause that negatively affects dairy animals, leading to substantial economic loss. The present study was aimed to analyze the effect of heat stress on cellular and molecular levels in dermal fibroblast of cattle and buffaloes. Primary fibroblast culture was established using ear pinna tissue samples of cattle (*Bos indicus*) and riverine buffaloes (*Bubalus Bubalis*). The cells were exposed to thermal stress at 42°C for 1 h and subsequently allowed to recover and harvest at 37°C at different time points (0, 2, 4, 8, 16, and 24 h) along with control samples. Different cellular parameters viz., apoptosis, proliferation, mitochondrial membrane potential (Δ*Ψ*_m_), oxidative stress, along with expression pattern of heat responsive genes and miRNAs were determined. Cell viability and proliferation rate of heat-stressed fibroblasts decreased significantly (*P* < 0.05) albeit to a different extent in both species. The cell cytotoxicity, apoptosis, production of reactive oxygen species, and Δ*Ψ*_m_ increased more significantly (*P* < 0.01) in heat stressed fibroblasts of buffalo than cattle. The pattern of heat shock proteins, inflammation/immune genes, and heat responsive miRNA showed differences in induction of their expression level in buffalo and native cattle fibroblasts. Conclusively, finding indicates that heat stress induces more profound impact on buffalo fibroblasts than native cattle fibroblasts. The differential response of cellular parameters, HSP genes, and miRNA expression could be due to better adaptive capacity of skin fibroblast of *Bos indicus* cattle in comparison with riverine buffaloes.

## Introduction

Environmental heat stress imposes a significant burden on dairy animal production across the world by adversely affecting their production and reproduction ability. The concern has increased even more in recent years with the realization of global warming impact on the environment [1]. Animals experiencing heat stress tend to reduce their heat production by limiting feed intake, with subsequent negative effects on growth performance, causing huge economic loss to the dairy industry. A combination of various environmental factors, physical, and genetic characteristics of the animal affect the thermal imbalance of animals [2]. Apart from management strategies to combat heat stress, exploring the genetic differences and molecular responses will be significant for the improvement of thermo-tolerant ability on a long-term basis. The substantial differences in heat tolerance ability both at species and breed level are well documented [3,4]. Furthermore, there are several anatomical and physiological factors that control the thermoregulation ability of animals. Properties of skin, length of hairs, the relationship between surface area per unit body weight, and metabolic heat production are some of the well-known factors that influence heat stress response [5].

Skin being the first defense mechanism of the immune system allows itself to respond and resist to the hostile environment of temperature, humidity, and radiations. Dermal fibroblasts are one of the major constituents of skin, contributing to homeostasis of the skin, and also involved in various physio-pathological conditions. They are spindle-shaped cells and actively involved in interactions with other cell types by cell–matrix or direct cell interactions [6]. Considering these characteristics, dermal fibroblast could be used as a cellular model to study the impact of heat stress. Further, it has been reported that differences in functional and morphological characteristics of skin could help explain differences in heat tolerance among species and breeds [1,4,6]. Although few studies reported the response of fibroblast to heat stress, still the information on the cellular and transcriptional level in livestock especially dairy animals is meager. India is a home to several best zebu cattle (*Bos indicus*) and riverine buffalo (*Bubalus bubalis*) breeds well-known for their hardiness and survival under stressed conditions. Both cattle and riverine buffaloes are major contributors to milk production and play a significant role in maintaining the economy of dairy sectors as well as for farmers. It would be imperative to study the effect of heat stress on these two important dairy species. The role of fibroblast in thermos-tolerance ability in terms of cellular response and post-transcriptional changes is not fully elucidated in livestock species. In the past, attempts were made to better understand the molecular aspects of heat stress response in livestock. Several studies indicated that exposure to heat stress reduced cell proliferation and induced cell death [7–9]. These reports have also highlighted the potential role of heat shock proteins (HSPs) in maintaining cellular homeostasis and considered as primary elements in cellular stress responses [10,11].

Currently, miRNAs are also being explored as potential bio-markers of the health state of animals under diseased or stress state. During the cellular stress responses, miRNA plays a key role in a wide array of biological processes by regulating the abundance of gene expression. Only a few reports are available in livestock species describing the potentially expressed miRNA under heat stress [12,13]. This particular study was aimed to evaluate the effect of heat stress on cellular, transcriptional, and post-transcriptional changes in dermal fibroblast cells of Sahiwal cows (native cattle) and Murrah buffaloes (riverine buffaloes) to assess their cellular tolerance.

## Materials and methods

### Experimental animals

Six healthy animals (three each of Sahiwal cows and Murrah buffaloes) ranging from 1 to 2 years of age group were selected from an organized herd of National Dairy Research Institute (NDRI), Karnal for experiments. All these animals were maintained under general management practices at the Institute. At the time of actual experiment, all animals were clinically healthy and free from any physical or anatomical abnormalities. The experiment was approved by the Institutional Animal Ethics Committee (IAEC). Norms regarding the ethical treatment of animals during the whole operation were strictly followed.

### Primary skin fibroblast cell culture

Skin biopsy specimens were aseptically taken from ear margin (about 1 cm^2^ in size) from three animals of Sahiwal cows and Murrah buffaloes and collected into separate tubes containing DMEM medium (Gibco, U.S.A.) with ampicillin (100 U/ml) and streptomycin (100 µg/ml). The samples were rinsed and chopped into 1 mm^3^ pieces, which were seeded into the medium in a tissue culture flask (Corning, U.S.A.) containing DMEM (Gibco, U.S.A.) +10% fetal bovine serum (Hyclone, U.S.A.) incubated at 37°C with 5% CO_2_. Cells were harvested when they reached 80–90% confluence and were divided into prepared culture flasks at ratios of 1:2.

### Heat treatment of skin fibroblast cells

The cow fibroblast (CFb) and buffalo fibroblast (BFb) after third passages were exposed to heat stress at 42°C for 1 h and thereafter allowed to recover at 37°C and harvested at different time intervals (2, 4, 8, 16, and 24 h) by trypsinization. The untreated cells (control) were kept at 37°C throughout the course of the study and were harvested at the same time points corresponding to the treated samples. The cells were subjected for determination of various cellular parameters including viability, cytotoxicity apoptosis, and changes in mitochondrial membrane potential. The cells were kept in ice-cold Trizol (Invitrogen, Carlsbad, California) for extraction of total RNA.

### Flow-cytometry based cellular assay

#### Cell viability and apoptosis

Cell viability and apoptosis were determined using Muse™ Annexin V and Dead Cell Kit (Millipore) according to the manufacturer’s protocol on the Muse Cell Analyzer (Millipore, Bedford, MA, U.S.A.). The percentage of cells undergoing apoptosis after heat treatment was calculated by summing up the number of cells from late and early apoptotic quadrants of the histograms. The Muse™ Annexin V & Dead Cell Assay utilizes Annexin V to detect phosphatidylserine (PS) on the external membrane of apoptotic cells and 7-AAD (7- amino-actinomycin D) was used as an indicator of cell membrane structural integrity.

#### Oxidative stress and mitochondrial membrane potential

Oxidative stress was measured by Muse® oxidative stress kit (Millipore) in a Muse Cell Analyzer (Millipore, Bedford, MA, U.S.A.). Briefly, 1 × 10^6^ cells were incubated with Muse® oxidative stress working solution at 37°C for 30 min and then mixed thoroughly before being analyzed on the cell analyzer. Changes in mitochondria membrane potential (Δ*Ψ*m) was measured using Muse™ MitoPotential assay kit (Millipore). This assay helped to differentiate four different populations of cells: live cells with the depolarized mitochondrial membrane, live cells with the intact mitochondrial membrane, MitoPotential+/7-AAD−; dead cells with the depolarized mitochondrial membrane, MitoPotential+/7-AAD+; and dead cells with the intact mitochondrial membrane, MitoPotential−/7-AAD+.

#### Cell proliferation and cytotoxicity determination

Cells were plated at 1 × 10^4^ cells per well in 96-well plates and heat stress was given as described above. Cell proliferative response of BFb and CFb in unstressed and heat stressed cells was assessed by CyQuant Proliferation Kit (Molecular Probes). After heat stress, the contents of the assay wells were removed. Subsequent cell labeling with the CyQuant reagent was according to the manufacturer’s instructions. Fluorescence intensity of each sample was measured using a fluorescence microplate reader with excitation at ∼485 nm and emission detection at ∼530 nm.

The cytotoxicity was assessed by measurement of LDH released into the medium in heat stressed and unstressed cells. At the end of treatment from each time point, 100 μl of the medium was transferred to a 96-well plate and the LDH assay was performed using LDH Cytotoxicity Assay Kit (Cayman, Ann Arbor) following the manufacturer’s instructions. To determine LDH activity, the absorbance at 490 nm was taken and cytotoxicity was calculated in terms of fold LDH released.

### mRNA and microRNA expression analysis

Total RNA including microRNA from heat stressed and unstressed CFb and BFb was extracted using ice-cold Trizol (Invitrogen, Corp., CA) followed by column purification and RNase free DNase digestion using RNeasy Mini Kit (Qiagen, Germany). The RNA concentration and purity were measured using a NanoDrop ND-1000 spectrophotometer (NanoDrop Technologies) and stored at −80°C until further usage. Complementary DNA (cDNA) was synthesized using Revert Aid First Strand cDNA Synthesis Kit (Fermentas, U.S.A.) according to the manufacturer’s instructions. From the purified RNA, cDNA was reverse transcribed using Mir-X™ miRNA First-Strand Synthesis kit (Takara- Clonetech). The mixture was incubated at 37°C for 1 h and then terminated at 85°C for 5 min to inactivate the enzymes. The first strand cDNA of miRNA and mRNA was diluted 1:150 and 1:4 (v:v) respectively, with DNase/RNase free water. In the present study, expression of 10 heat responsive mature miRNAs viz., miRNA-19a, miRNA-19b, miRNA-26a, miRNA-27b, miRNA-30a-5p, miRNA-146a, miRNA-146b, miRNA-199a-3p, miRNA-1246 and miRNA-345-3p selected from literature (Zheng et al., 2014) were evaluated in heat stressed and unstressed CFb and BFb. The primer details for each miRNA are given in Table 1. The reactions were performed with amplification conditions: 2 min at 50°C, 10 min at 95 °C, 40 cycles of 10 s at 95°C (denaturation) and 1 min at 60°C (annealing + extension). A dissociation protocol with an incremental temperature of 95°C for 60 s, 55°C for 30 s and 95°C for 30 s was used to investigate the specificity of qPCR reaction and the presence of primer dimers. Further expression data were normalized with three internal control genes: U6 snRNA, S18, and β5s-RNA and compared using first derivative method [14].

**Table 1:** Details of annealing temperature and primers sequences of targeted miRNAs

S.No.	miRNAs/Reference genes	Primer sequences	*T*_a_ (°C)
**microRNAs**			
1.	miRNA-19a	F:5′-CGGCGGTGTGCAAATCTAT-3′	60
2.	miRNA-19b	F:5′-TGTGCAAATCCATGCAAAACTG-3′	60
3.	miRNA-26a	F:5′-GTTCAAGTAATCCAGGATAGGCT-3′	60
4.	miRNA-27b	F:5′-CGGCTTCACAGTGGCTAAGTTCT-3′	60
5.	miRNA-30a-5p	F:5′-CGGTGTAAACATCCTCGACTGG-3′	60
6.	miRNA-146a	F:5′-GCGGCGGTGAGAACTGAAT-3′	60
7.	miRNA-146b	F:5′-CGCCGGTGAGAACTGAAT-3′	60
8.	miRNA-199a-3p	F:5′-CGGACAGTAGTCTGCACATTGG-3′	60
9.	miRNA-345-3p	F:5′-GCCTGAACTAGGGGTCTGGAG-3′	60
10.	miRNA-1246	F:5′-GGAATGGATTTTTGGAGCAGG-3′	60
**Reference genes**			
11.	β-5s RNA	F:5′-CTCGTCTGATCTCGGAAGCTAA-3′	60
12.	5S rRNA	F:5′-GCCCGATCTCGTCTGATCT-3′	60
13.	U6 snRNA	F:5′-TTATGGGTCCTAGCCTGAC-3′	60
14.	S18	F: 5′-CACCGAGGATGAGGTGGA-3′	60

Additionally, expression of eight heat-responsive genes viz., *HSP70, HSP90, HSP60, HSP40, Bax, BCl2, Fas*, and *TNF-α* were also evaluated in stressed and unstressed fibroblast cells of both the species. The primer details for each mRNA are given in Table 2. Each reaction was performed using 4 µl diluted cDNA combined with 6 µl of a mixture composed of 2 µl 5× EvaGreen (Solis Biodyne), 0.4 µl each of 10 pM forward and reverse primers, and 3.2 µl DNase/RNase-free water in a 96-well transparent plate (Thermo Fisher). The reactions were performed in a StepOne Plus instrument (ABI) along with dissociation curve.

**Table 2:** Details of primer sequences, annealing temperature (*T*_a_), slope, and PCR efficiency for the target genes

S.No.	Genes	Primer sequences (5′-3′)	*T*_a_ (°C)	Slope	PCR efficiency (%)
1.	*HSP70*	AACATGAAGAGCGCCGTGGAGG GTTACACACCTGCTCCAGCTCC	60	−3.260	103
2.	*HSP90*	CTGTCATCAGCAGTGGG ACATGCCAACAGGATCTAC	60	−3.161	107
3.	*HSP60*	CGACAACTTCTGCTGTTGTTA ATGATGCTATGCTTGGAGAT	60	−3.360	98
4.	*HSP40*	AACACAACGGGTATGGT AGCCAGGATCAGCCTTC	60	−3.125	109
6.	*Bax*	TTTGCTTCAGGGTTTCATCATCC CAGTTGAAGTTGCCGTCAGA	60	−3.382	98
7.	*BCl2*	ATGTGTGTGGAGAGCGTCAA CAGACTGAGCAGTGCCTTCA	60	−3.380	98
8.	*TNF-α*	AGGTGGCCCCTCCATCA GGCTACCGGCTTGTTACTTGA	60	−3.387	97
9.	*Fas*	AGTTGGGGAGATGAATGCTG CCTGTGGATAGGCATGTGTG	60	−3.214	105

### Statistical analysis

All experiments were carried out in triplicate to compare the differences at cellular and the expression (miRNA and mRNA) levels in heat stressed and unstressed fibroblast cells of native cows and riverine buffaloes. The delta *C*_T_ values were analyzed using the one-way ANOVA test followed by Tukey’s multiple comparison tests, and *P*-value of ≤ 0.05 was considered statistically significant. All data are presented as the mean ± SEM.

## Results and discussion

**Figure 1:**
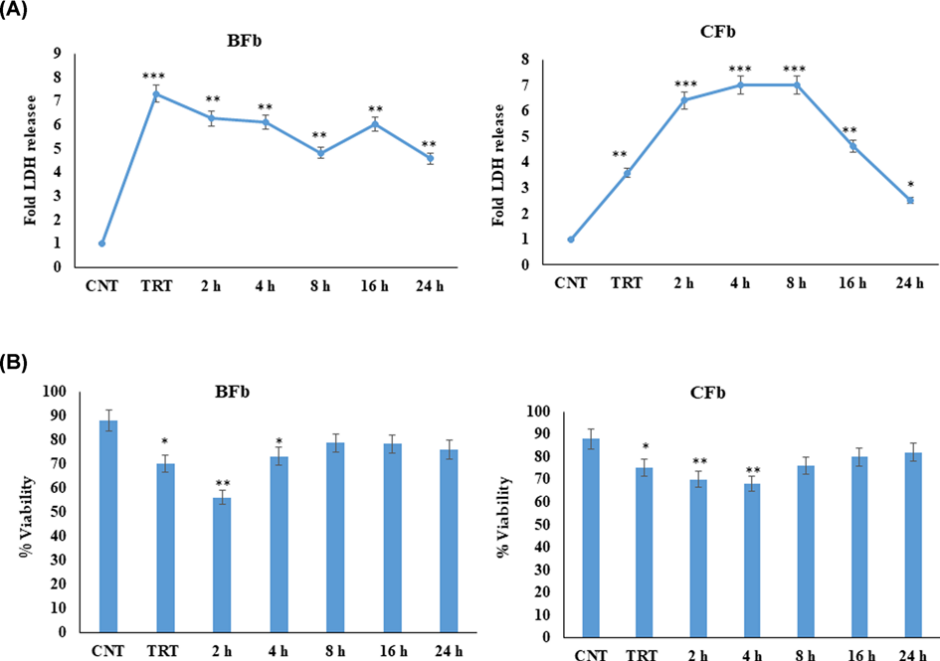
Cytotoxicity measured by fold LDH released (A) and cell viability (B) of buffalo fibroblast (BFb) and cattle fibroblast (CFb) after heat stress and at different recovery time points (post heat treatment) as compared with control (untreated); CNT-control, TRT- treated Data are presented as means of three separate experiments, error bars indicates SEM. The asterisk indicates a significant difference between control and respective sample; *P <0.05; **P <0.01; ***P <0.001.

Heat stress is known to induce mitochondrial apoptotic pathway in various cell types by inducing ROS, significant increase in cytochrome *c* release and significant loss in mitochondrial membrane potential [15–17]. In order to assess the relative cellular tolerance to heat stress, fibroblasts cells of Sahiwal cows (*Bos indicus*) and Murrah buffaloes (*Bubalus bubalis*) were exposed to thermal stress at 42°C for 1 h. The cells were harvested at different time points viz., 2, 4, 8, 16, and 24 h post heat stress. The cells designated as untreated were kept at 37°C throughout the course of experimentation. The LDH release, which is an indicator of cytotoxicity, increased significantly post heat stress and thereafter decreased continuously during the recovery period in BFb cells. However, CFb had maximum LDH release after 8 h of post-stress (Figure 1A). Though it was relatively lower in comparison with maximum cytotoxicity observed for BFb. These results suggested that heat stress exerts a cytotoxic effect on fibroblast of both native cattle and buffaloes; however, the extent of cytotoxicity was higher in BFb as compared with CFb indicating their higher susceptibility to heat stress. Similarly, the percentage of viable cells declined significantly in heat stressed BFb and CFb samples. In BFb, the percent viabilities with respect to control (unstressed) cells were 70%, 56%, 73%, while in CFb, the values were 75%, 70%, 68 % for 0, 2, and 4 h post heat stressed samples, respectively, (Figure 1B). A similar pattern of viability was reported by our group for buffalo mammary epithelial cells post heat stress [18]. The exposure to mild non-lethal elevated temperature is known to induce cellular stress in different cell types. Numerous studies in the past have revealed the direct relation between cellular response to heat stress and changes in viability/cytotoxicity parameters [18,19].

### Cell proliferation

**Figure 2:**
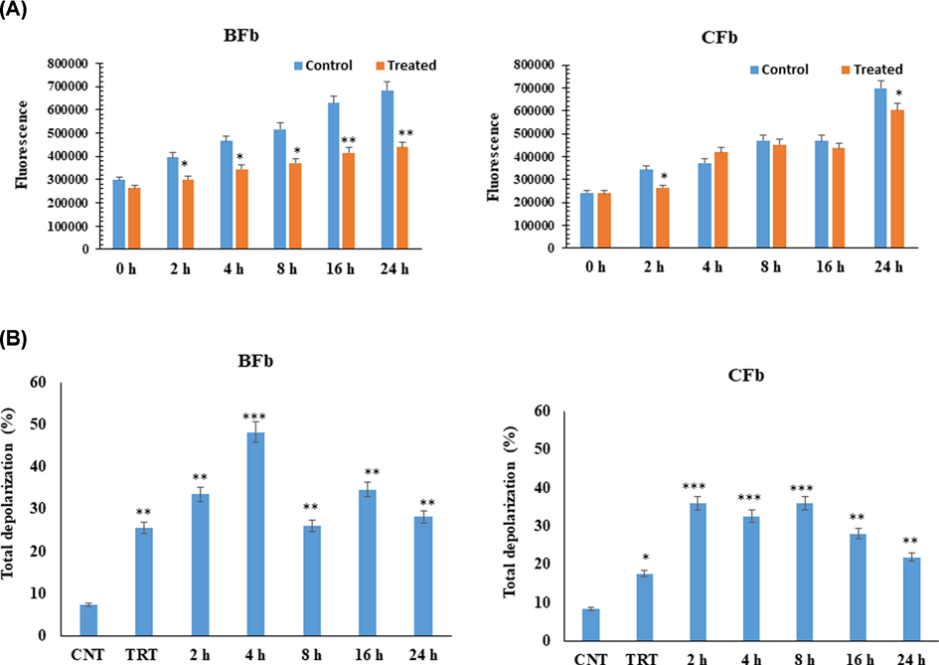
Cell proliferation response (A) and total depolarization of mitochondrial membrane potential (B) of buffalo fibroblast (BFb) and cattle fibroblast (CFb) post heat stress compared with control (untreated); CNT-control, TRT- treated Data are presented as means of three separate experiments, error bars indicates SEM. The asterisk indicates a significant difference between control and respective sample;*P < 0.05; **P < 0.01, ***P < 0.001.

The effects of heat stress on the proliferation rate of BFb and CFb are depicted in Figure 2A. For evaluating the cell proliferation rate, CyQuant proliferation assay (Invitrogen) that measures the cell number based on cell DNA content was used. The cell proliferation data of heat-stressed fibroblasts were compared with unstressed cells at different time points of the recovery phase. In both heat stressed BFb and CFb, the cell proliferation rate reduced significantly (*P* < 0.05). In BFb proliferation rate was decreased by 36% while in CFb it was reduced by 14% at 24 h. The decrease in cell proliferation efficiency in heat stressed fibroblast cells might be due to loss of plasma membrane potential. There has been accumulating evidence that heat stress inhibits cell proliferation mainly via G0/G1 and G2/M stage arrest [19]. Therefore, the adverse effect of heat on the proliferation of fibroblast could be associated with cell cycle stages. A similar observation was made in *in-vitro* heat stressed buffalo MECs [18].

### Mitochondrial membrane potential (Δ*Ψ*_m_)

In order to assess the effect of heat stress on mitochondrial dysfunction, depolarization of mitochondrial membrane potential (Δ*Ψ*_m_) was evaluated in CFb and BFb cells. The depolarization of Δ*Ψ*_m_ has always been regarded as a reliable predictor of mitochondrial dysfunction and cellular stress. The Δ*Ψ*m increased significantly (*P* < 0.05) in CFb as well as BFb immediately post heat stress. In BFb, the depolarization was increased by 3.4% (0 h), 4.5% (2 h), 6.5% (4 h), 3.5% (8 h),4.6% (16 h), 3.8% (24 h), while in CFb, it was increased by 2.1% (0 h),4.3% (2 h), 3.9% (4 h), 4.3% (8 h), 3.4% (16 h), 2.6% (24 h) post heat stress. The impact of heat stress on depolarization of Δ*Ψ*m was most significant (*P* < 0.001) between 2 and 4 h of post heat stress (Figure 2B). Though, the data indicated heat stressed induced depolarization of Δ*Ψ*m in fibroblast cells of both the species; however, the impact was more in BFb as compared to CFb (Figure 2B). It is generally perceived that the increase in membrane depolarization during heat stress leads to the opening of mitochondrial permeability transition pore and subsequently promote cell death [20,21]. Substantial evidence implicates mitochondria in apoptotic cell death. It is thought that proteins normally restricted to the mitochondrial intermembrane space, including cytochrome *c* and the 50-kDa apoptosis-inducing factor, are released to the cytosol where they initiate the apoptotic cascade [22]. Since the apoptosis of BFb and CFb cells initiated at 2 h after heat shock, it may be possible to say that the activation of caspase-3 may precede apoptosis in fibroblasts.

### Reactive oxygen species (ROS) production

**Figure 3:**
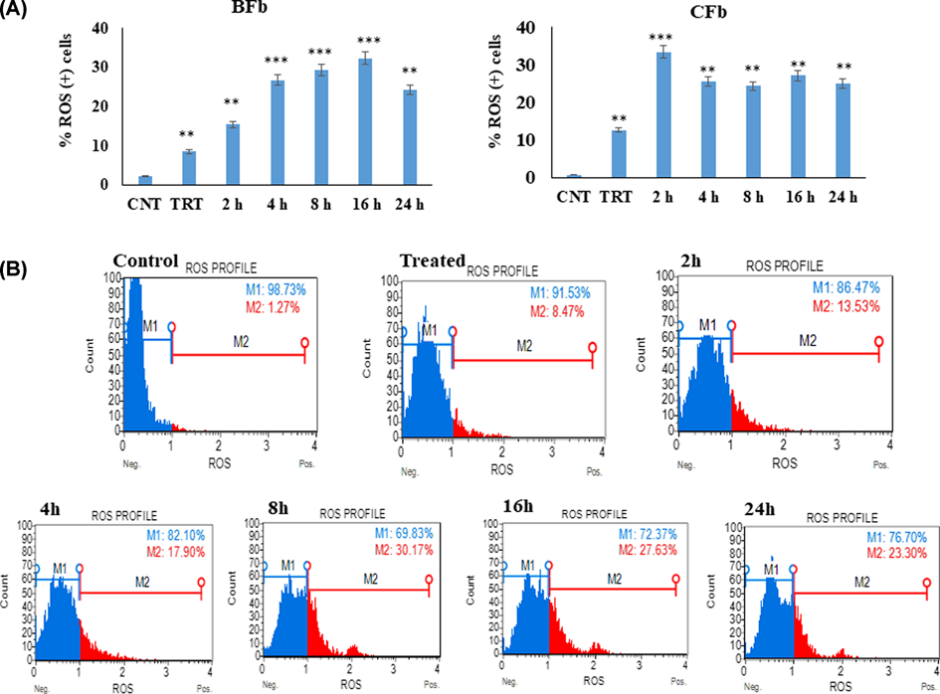
Oxidative stress in fibroblast post heat stress compared with control (untreated) (**A**) Buffalo fibroblast (BFb) and cattle fibroblast (CFb). CNT-control, TRT- treated. (**B**) Plots showing oxidative stress in buffalo fibroblast (BFb) post heat stress, M1 population is ROS (-) cells, M2 population is ROS (+) cells; these are representative plots of each time point of any one experiment. Data are presented as means of three separate experiments, error bars indicates SEM. The asterisk indicates a significant difference between control and respective sample; **P <0.01; ***P <0.001.

Previous studies have confirmed that heat stress-induced reactive oxygen species (ROS) generation promotes cellular apoptosis. However, the identity of the specific ROS involved in the process remained unclear [15,16]. In the present study, ROS (+) and ROS (-) cells were quantified using flow cytometry-based cell analyzer to determine the extent of oxidative stress in BFb and CFb post heat stress treatment. In BFb, the oxidative stress increased 3- and 6-fold immediately post heat stress (0 and 2 h; *P* < 0.01) compared with unstressed cells (Figure 3A,B). At later time points of recovery, the magnitude of oxidative stress further increased to 10-, 11.2-, and 13.6-fold at 4, 8, and 16 h, respectively (*P* < 0.001) before coming down to 8.8-fold post 24 h (*P* < 0.01) of the recovery phase. However, in CFb, the oxidative stress increased to 8-fold (0 h; *P* < 0.01) and 22.6-fold (2 h; *P* < 0.001) immediately post heat stress but decreased thereafter at 4, 8, 16, and 24 h of recovery phase compared with unstressed cells (Figure 3A). Our findings of increased oxidative stress in cells exposed to heat stress is in agreement with several previously published reports [23–25]. Many reports suggested that oxidative stress modulates miR biogenesis by affecting key molecules involved in miR maturation, such as the nuclear export factor exportin 5 and Dicer [26]. In addition, miRs themselves can be modified by oxidative stress, which alters their integrity, stability, binding affinity, and function [27].

### Induction of apoptosis

**Figure 4:**
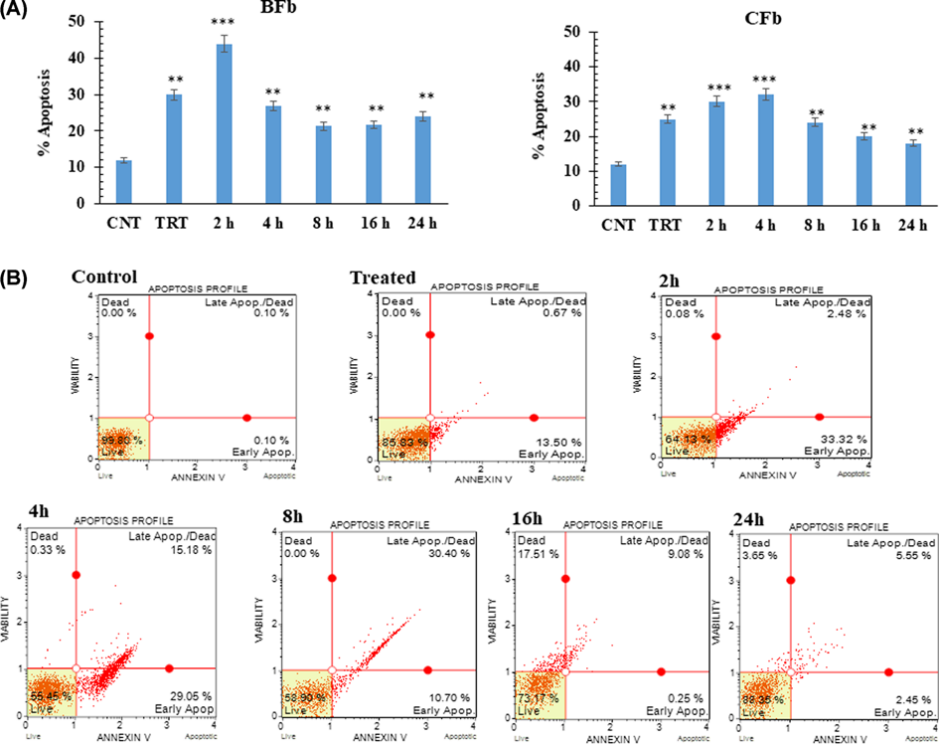
Analysis of apoptosis in buffalo and cattle firbroblast post heat stress (**A**) Buffalo fibroblast (BFb) and cattle fibroblast (CFb), compared with control (untreated). CNT -control, TRT - treated cells. (**B**) Representative flow cytometry scatter plots showing apoptosis rate in treated cells: the quadrants display distribution of live (bottom left), early apoptotic (bottom right), apoptotic (upper right) and dead (upper left) cells. Data are presented as means of three separate experiments, error bars indicates SEM. The asterisk indicates a significant difference between control and respective sample; *P <0.05; **P <0.01.

Accumulating evidence proves that induction of ROS due to heat stress can stimulate cell death and tissue injury, and that apoptosis plays a key role during this process. To assess the impact of heat stress on induction of apoptosis in BFb and CFb cells, Annexin V assay was used to determine the percentage of apoptotic and dead cells. Early in the apoptotic pathway, molecules of PS are translocated to the outer surface of the cell membrane where Annexin V can readily bind them. The analysis yielded four types of cell populations; viable cells: Annexin V (-) and 7-AAD (-), early apoptotic cells: Annexin V (+) and 7-AAD (-), late apoptotic/ dead cells: Annexin V (+) and 7-AAD (+) and dead cells: Annexin V (-) and 7-AAD (+). At 0 and 2 h post heat stress, 30% (*P* < 0.01) and 45% (*P* < 0.001) of BFb cells were found to be Annexin-V positive indicating induction of apoptosis immediately after heat stress (Figure 4A,B). At later stages of recovery phase, the BFb undergoing early apoptosis were 28% at 4 h (*P* < 0.01), 20% at 8 h (*P* < 0.01), 20% at 16 h (*P* < 0.01) and 25% at 24 h (Figure 4A). Similarly, the percentage of cells undergoing early apoptosis in CFb were; 25% at 0 h (*P* < 0.01), 30% at 2 h (*P* < 0.001), 35% at 4 h (*P* < 0.001), 30% at 8 h (*P* < 0.01), 20% at 16 h (*P* < 0.01) and 18% at 24 h (*P* < 0.01) post heat stress. The proportion of late apoptotic/dead cells increased with increase in time points post heat stress (Figure 4A). Similarly, high production of ROS and mitochondrial membrane depolarization was observed at 2–4 h post heat stress. Based on the present analysis and those reported by other workers, it is suggested that activation of the apoptotic pathway during heat stress could be the major cause of cell death [28,29]. Similar to our results, previous studies also reported that hyperthermia at 42–46°C for 30–60 min induces cell death [30,31].

The findings indicated that 1 h of heat stress given to fibroblast cells of both the species resulted in decreased cell viability and cell proliferation rate, while increased cytotoxicity, apoptosis and ROS levels. Overall, the fibroblast cells of Sahiwal cows has the lesser impact of heat stress on cellular functions in comparison to Murrah buffaloes. The data presented here provide evidence that Sahiwal cow’s fibroblasts have better cellular tolerance than Murrah buffaloes’ fibroblasts to heat stress. The heat shock treatment given to both BFb and CFb cells did not have an irreversible impact in both the cell types, as within few hours of the post-stress recovery phase, the cell proliferation rates started increasing though gradually. Similarly, the results for mitochondrial membrane potential, oxidative stress, and cellular apoptosis further indicated to the fact that the heat stress treatment given to both the cells was sub-lethal. The enhanced oxidative stress in heat stressed BFb and CFb suggested to the fact that skin cells undergo extensive changes to protect itself from increased oxidative stress probably through activation of its antioxidant machinery [32].

### Transcriptional changes in heat stressed fibroblasts of CFb and BFb

**Figure 5:**
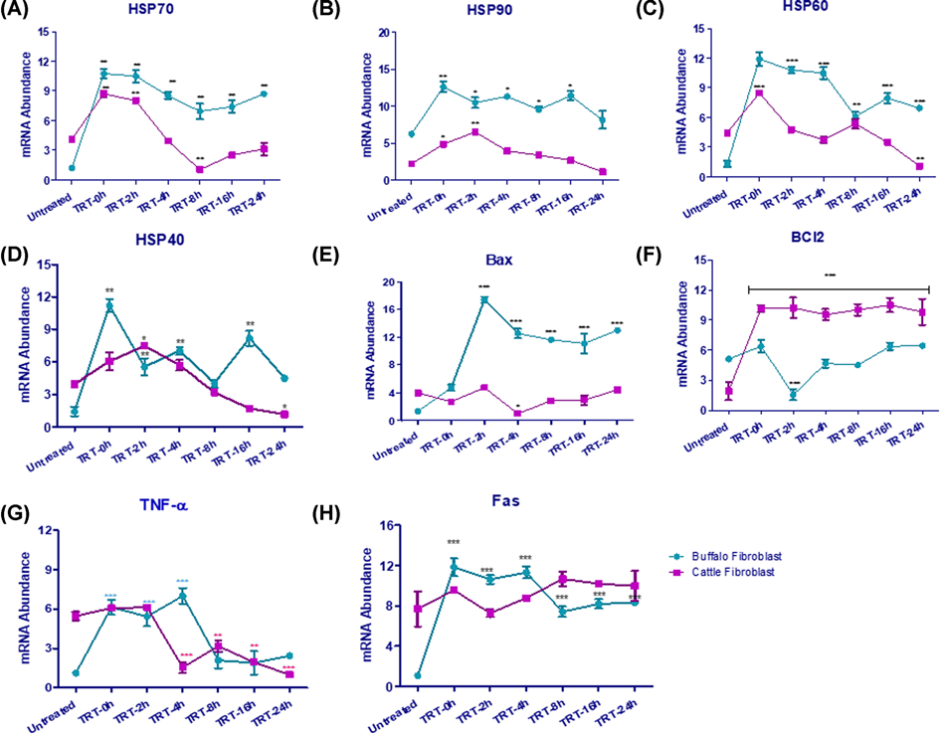
Gene expression analysis in buffalo and cattle fibroblast post heat stress as compared with control (untreated) cells (**A**) HSP70, (**B**) HSP90, (**C**) HSP60, (**D**) HSP40, (**E**) Bax, (**F**) BCl2, (**G**) TNF-α, (**H**) Fas. Data are presented as means of three separate experiments, error bars indicates SEM. The asterisk indicates a significant difference between control and respective sample. TRT- treated; *P<0.05; **P<0.01; ***P<0.001.

To investigate the impact of transcriptional changes, a panel of genes associated with heat shock response, apoptosis, inflammatory response, and signaling processes were evaluated in heat stressed and unstressed CFb and BFb. The PCR efficiency values (ranging from 97 to 109%) of target genes indicated the quality of qPCR expression data (Table 2). For normalization of target gene *C*_T_ values, the geometric mean of four reference genes (*GAPDH, RPS23, RPL4*, and *EEF1A1*) were used. As expected, the expression of heat shock protein genes *viz. HSP 70, HSP 90, HSP40*, and *HSP 60* increased significantly (*P* < 0.05) though to a different extent in heat stressed CFb and BFb cells (Figure 5A–D). For example, the expression of *HSP70* mRNA induced significantly (*P* < 0.01) in BFb at all-time points post heat stress (0, 2, 4, 8, 16, and 24 h) (Figure 5A). In BFb, the *HSP70* transcript increased to 8.88-, 8.67-, 7.05-, 5.74-, 6.12-, and 7.12-fold after 0, 2, 4, 8, 16, and 24 h of heat stress respectively. In CFb as well, *HSP70* expression increased significantly (*P* < 0.01) after 0 and 2 h of heat stress and then reduced to non-significant (*P* > 0.05) level at later time points (4, 8, 16, and 24 h). The extent of induction of *HSP70* at early time points was lower in CFb in comparison with BFb (Figure 5A). Similarly, the *HSP90*, mRNA also increased significantly in BFb (*P* < 0.05) after 0, 2, 4, 8, and 16 h of heat stress (Figure 5B). In CFb, the induction was significant (*P* < 0.01) only at 2 h time point post heat stress. At 0 and 4 h only tendencies (*P* < 0.05) of the increase was observed before returning close to the basal level at 8, 16, and 24 h. The other two HSPs; *HSP60* and *HSP40*, also induced significantly (*P* < 0.01) after 0, 2, 4 h of heat stress in BFb (Figure 5C,D). The level of induction for both *HSP60* and *HSP40* genes at 8 and 24 h after heat stress was relatively lower in comparison with other time points. On the other hand, in CFb, the significant (*P* < 0.05) induction for these two genes was observed only at a single time point after heat stress; 2 h for *HSP40* and 0h for HSP60 post heat stress (Figure 5C,D). In the present study, overall, the extent of induction of HSP genes in CFb was consistently lower in comparison to BFb. This could be attributed to the difference in skin fibroblast characteristics of the two dairy species and their differential responsiveness to heat stress exposure [33]. Interestingly, the response of BFb and CFb to thermal stress was quite similar in the sense that induction in the expression of *HSP70, HSP90, HSP60*, and *HSP40* genes happened during early hours of heat stress (0, 2, and 4 h) and subsequently, their expression declined at later stages (8–24 h). In the past, several studies have utilized the transcriptional changes of HSP genes as an indicator to evaluate the ability of cellular tolerance in different cell types. It is now a well-accepted notion that increased expression of HSP gene family is essential to provide tolerance against thermal stress and enhance the cell survivability [34]. Similar trend in expression pattern of HSP genes was reported in other cell types (leukocytes/lymphocytes/MECs) [11,18,34,35]. Molle et al. [36] have demonstrated that HS treatment of mice leads to a strong induction of *HSP70* in several organs and confers significant protection against lethality induced by *TNF-α*. In addition, they have shown that mice deficient in the inducible hsp70.1 gene were no longer protected by HS treatment, indicating that HSP70 is at least necessary for the conferred protection induced by whole-body HS of mice. A number of studies reported that deteriorating cellular effects of free radicals species induces expression of heat-responsive genes to protect biological systems from oxidative stress damage [37–39]. Similarly, in present study, the expression of HSPs increased as ROS production increased post heat stress. Furthermore, several hypotheses have been formulated that suggest that heat stress can indirectly activate heat shock factor which is a regulator of HSPs, via the action of ROS [40].

Expression of pro-apoptotic BCl2 Associated X, Apoptosis Regulator (*Bax*) increased significantly (*P* < 0.001) in BFb, while the increase in its expression in CFb was non-significant (*P* > 0.05) after heat stress (Figure 5E). In contrast, the expression of the anti-apoptotic gene (*Bcl2*) did not show significant changes in heat stressed BFb, while its expression increased significantly (*P* < 0.01) at all-time points after heat stress in CFb (Figure 5F).Relative concentrations of anti- and pro-apoptotic genes determine whether particular cell types under study will survive or undergoes apoptosis in response to external stress [41]. Similar to HSPs and proapoptotic genes, the expression of *TNF-α*; major pro-inflammatory cytokine also increased substantially (*P* < 0.01) immediately post stress (0, 2, and 4 h) in BFb and then returned to non-significant level during later time points. In contrast, the increase in its expression in CFb was non-significant during early time points (0 and 2h) post stress in comparison to unstressed cells. The induction of *TNF-α* mRNA post heat stress was relatively higher in BFb as compared with CFb (Figure 5G). TNF-*α* and the Fas ligand are cell-death mediators that act by binding to their responsive receptors, two TNF receptors (TNFR) TNFR1 and TNFR2, and Fas, respectively, and induce apoptosis in a variety of cell types [42]. Similarly, in our study, we found that higher expression of TNF-*α* in BFb caused the higher apoptosis rate as shown in Figure 4. Consistent with our results, DeMeester et al. have demonstrated that induction of HS in cells primed by inflammation can precipitate cell death by apoptosis, whereas a prior heat shock stress can protect the cell against inflammatory stress both *in vitro* and *in vivo* [43]. They called this seemingly paradoxical ability of HS to induce cytoprotection and cytotoxicity the ‘heat shock paradox’. Generally, *TNF-α* induced by stress is known to further activates *HSP70* expression through *TNF-α* 1 receptor [44]. In addition, the *Fas* gene expression pattern was also evaluated in both BFb and CFb cells post heat stress. The protein encoded by this gene is a member of the TNF-receptor superfamily. This receptor contains a death domain. It has been shown to play a central role in the physiological regulation of programmed cell death. We observed that *Fas* gene expression was significantly (*P* < 0.001 for 0, 2, and 4 h; *P* < 0.01 for 8, 16 and 24 h) affected by heat stress in BFb but not in CFb (Figure 5H). *Fas* gene expression is strongly regulated by heat shock factor 1 (HSF1) which is a primary heat shock transcription factor [45]. Also, HSF-1 mediated induction of Fas ligand gene expression in activated T cells [46]. In the present study, our purpose was to determine the expression kinetics of several heat-responsive genes in fibroblast cells of native cattle and riverine buffaloes in response to mild thermal stress. The data suggested substantial changes in the pattern of expression of genes known to be heat responsive, apoptotic, pro-inflammatory cytokines; albeit to a varied extent in cattle and buffalo fibroblast cells indicating the species difference in response to heat stress.

### Post-transcriptional changes in heat stressed fibroblasts of CFb and BFb

**Figure 6:**
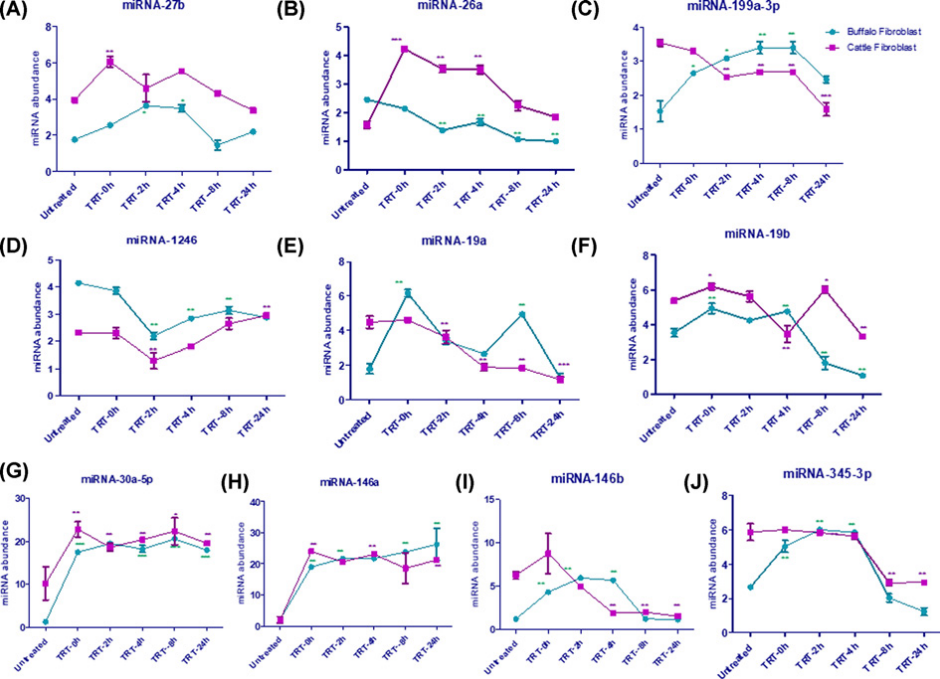
miRNA expression analysis in buffalo and cattle fibroblast post heat stress compared with control (untreated) (**A**) miRNA-27b, (**B**) miRNA-26a, (**C**) miRNA-199a-3p, (**D**) miRNA-1246, (**E**) miRNA-19a, (**F**) miRNA-19b, (**G**) miRNA-30a-5p, (**H**) miRNA-146a, (**I**) miRNA-146b, (**J**) miRNA-345-3p. Data are presented as means of three separate experiments, error bars indicates SEM. The asterisk indicates a significant difference between control and respective sample. TRT- treated; *P <0.05; **P <0.01; ***P <0.001.

Ten mature microRNAs (*miR-26a, miR-27b, mir-1246, miR-19a, miR-19b, miR-345-3p, miR-30-5p, miR-146a, miR-146b*, and *miR-199a-3p*) evaluated in heat stressed and unstressed BFb and CFb were selected on the basis of their responsiveness to heat stress in Holstein cows [12]. The up-regulation of majority of the microRNAs; though the degree of expression varied in skin fibroblast cells of two dairy species indicated their responsiveness to heat stress. In BFb, most of the miRNAs, except, *miR-26a, miR-1246* showed immediate induction in expression after heat stress (Figure 6A–J). In CFb as well as most of the miRNA except *miR-199a-3p, miR-1246*, and *miR-19a* showed induction in their expression after heat stress. However, the pattern and extent of expression of most of the miRNAs, (except miR-1246, miR-30a-5p, *miR-146a*, and *miR-345-3p*) were quite distinct in BFb and CFb after heat stress (Figure 6). For example, expression of *miR-199a-3p* induced significantly at 0 h (*P* < 0.05), 2 h (*P* < 0.01), 4 h (*P* < 0.01), and 8 h (*P* < 0.01) before returning close to basal (unstressed) at 24 h after heat stress (Figure 6C). On the other hand, the expression kinetics of *miR-199a-3p* was quite different in CFb, as its expression decreased at different time points (2 h *P* < 0.01; 4 h *P* < 0.01; 8 h *P* < 0.01; and 24 h *P* < 0.001) after heat stress (Figure 6C). The target gene for *miR-19a* has been reported to be *HSPA1A*. Similarly, *miR-19a* expression remained elevated significantly (*P* < 0.01) in BFb at 0, 2, and 8 h of heat stress. However, its expression reduced close to basal values at 4 and 24 h (Figure 6E). In contrast, its pattern of expression was quite distinct than BFb as its expression induced marginally at 0 h after heat stress and subsequently declined at all-time points. Similar to *miR-19a*, expression of *miR- 19b* also induced in CFb at 0 h (*P* < 0.05) and declined at 2, 4, and 24 h (Figure 6F). However, in contrary to *miR 19-a*, its expression was significantly (*P* < 0.01) up at 8 h after heat stress. For both *miR-19a/19b*, the target genes are *HSPBAP1, DNAJB1, HPX*/ *HSPBAP1, DNAJB1, HPX, APOA2, NOD2* whose functions are related to response to stress, oxidative stress, and immune response [12]. Since, these miRNA regulate genes from different functional categories, to understand their distinct pattern of expression during heat stress, would be really prudent to understand their function in large ruminant in future experimentation.

Similarly, *miR-146a* got significantly (*P* < 0.01) induced in both BFb and CFb immediately post heat stress (0 h) and remained up-regulated during other time points as well (2, 4, 8, 24 h). This miRNA showed similar pattern of expression in fibroblast cells of both the species (Figure 6H) indicating, the involvement of *miR-146a* in heat stress associated cellular response. Similar to *miR-146a, miR-146b* also remained elevated significantly (*P* < 0.01) at 0, 2, 4 h after heat stress in BFb (Figure 6I). However, in CFb, it showed non-significant induction at 0 h after heat stress and subsequently reduced its expression significantly (*P* < 0.01) at other time points. For both these (*miR-146a/b*), the target gene is toll-like receptor 4 (*TLR4*) which is known to be involved in innate immunity. The *TLR4* basic function is to activate the immune response during stress mediated through exposure to high temperature or the stimulation with lipopolysaccharide. Up-regulation of *miR-146a* after lipopolysaccharide (LPS) challenge on monocytes and suggested an anti-inflammatory role of this particular miRNA [47]. However, the discord in the expression of both these miRNA in BFb and CFb requires further investigation. The *miR-1246*, another miRNA associated with TLR regulation was evaluated in the skin fibroblast of cattle and buffalo. Its expression pattern was similar in both BFb and CFb (Figure 6D). Interestingly, both these miRNAs expression declined immediately after heat stress (0 and 2 h) and then started rising gradually from 4h to 24h. Like, *miR-146a/b*, this miRNA has also been suggested to have an influence on the immune system [12].

Similar to *miR-146a, miR-30a-5p* showed a similar expression pattern in BFb and CFb cells. Its expression induced significantly after heat stress and remained elevated significantly (*P* < 0.01, 0.001) at most of the time points post heat stress (Figure 6G). The target genes for this particular miRNA are reported to be *PLA2R1, PICEN*, with defined GO terms as response to oxidative stress and response to stress, respectively [12]. Recently, *miR-30a-5p* was reported to lessen inflammatory responses and oxidative stress by targeting Neurod 1 through MAPK/ERK signaling. *MiR-30a-5p* ameliorates injury-induced inflammatory responses and oxidative stress by targeting Neurod 1 through MAPK/ERK signaling [48]. Interestingly, *miR-27b* and *miR-26a* showed substantially higher expression level in CFb as compared to BFb both before and after heat stress (Figure 6A,B). Their expression induced at 0 h time point post stress but declined rapidly at subsequent time points after heat stress. The target predicted for *miR-26a* and *miR-27a* were *HSPA8, CD38, PRKCB*, and *TP53, PLA2R1, HSPCB, PSEN2, FYN, THY1, BCL10*, respectively.

We found that expression of *miR-345-3p* rose significantly (*P* < 0.01) in BFb after heat stress and reached to a level similar in CFb at 2 and 4 h before declining rapidly at 8 and 24 h after stress in both the fibroblasts (Figure 6). Similar to our results, its expression was significantly (*P* < 0.01) high in the serum of heats stressed Holstein Frisian cows [12]. M*iR-345-3p* known to be associated with energy metabolism and stress pathway [49]. The targets for this miRNA are *NOD2, THY1* genes, which are known to be a positive regulator of B- and T-cell activation, respectively. The panel of miRNAs evaluated in the present work showed substantial changes in expression at different time points after heat stress, indicating their potential role in regulating heat stress response. The difference in the expression pattern of selected mRNAs and miRNAs in skin fibroblast of two species strongly indicate to the fact that heat stress response differently and could be the reason for the difference in cellular tolerance of Sahiwal cows and Murrah buffaloes to heat stress. However, the exact reason for the more responsive character of BFb to heat stress is unclear and needs further study. The present study has thus helped to provide information on the expression pattern of selected mRNAs and miRNAs in heat stressed fibroblasts of cattle and buffaloes. In the future, such information will be helpful to link miRNAs expression and heat stress response of cattle and buffaloes.

To our knowledge, this is the first profiling of panel of heat stress responsive miRNAs in skin fibroblast cells of Indian native cattle and riverine buffaloes. These miRNAs may have stress. We found upregulation of *miRNA-30a-5p, miRNA-146a, miRNA-146b, miRNA-19b* while down-regulation of *miRNA-199a-3p, miRNA-1246, miRNA-26a* post heat shock. Variations in the expression level of these miRNAs at different time point post heat stress indicate about their importance during cellular response to heat stress. The prediction of target genes and network analysis of these miRNA could be interesting in future studies for better understanding of the mechanism of heat stress regulation. In the future, it would be interesting to evaluate some of these miRNAs in heat stress response by studying their specific roles in regulating gene networks and molecular pathways they represent.

In conclusion, our findings demonstrated that how *in vitro* heat stress could affect the cellular and molecular response of skin fibroblast cells of two major dairy species by undertaking detailed investigations on parameters like viability, cytotoxicity, oxidative stress, depolarization of mitochondrial membrane potential, cell apoptosis/death, and evaluation of genes/miRNAs associated with heat stress response. The data generated in the present study has given a strong indication that fibroblast cells of native Indian cows (Sahiwal cattle breed) has superior cellular tolerance in comparison to that of riverine buffaloes (Murrah buffalo breed). These findings suggest that in future skin fibroblast cells could be utilized as an important cellular model to understand the heat tolerance ability of dairy animals

## Abbreviations

BFb: Buffalo fibroblast
CFb: Cattle fibroblast
HSP: Heat shock protein
LDH: Lactate dehydrogenase
miRNA: Micro-RNA
ROS: Reactive oxygen species
TNFR: TNF receptors
